# Experiences, Enablers, and Challenges in Service Delivery and Integration of COVID-19 Vaccines: A Rapid Systematic Review

**DOI:** 10.3390/vaccines11050974

**Published:** 2023-05-11

**Authors:** Sarah Nabia, Chizoba Barbara Wonodi, Alba Vilajeliu, Sabine Sussman, Katharine Olson, Rianna Cooke, Krishna Udayakumar, Claire Twose, Nwamaka Ezeanya, Adewumi Adetola Adefarrell, Ann Lindstrand

**Affiliations:** 1 USAID’s MOMENTUM Country and Global Leadership, International Vaccine Access Center (IVAC), Johns Hopkins University, Baltimore, MD 21231, USA; snabia1@jhu.edu; 2 Department of Immunization, Vaccines & Biologicals (IVB), World Health Organization, WHO, 1211 Geneva, Switzerland; avilajeliu@who.int (A.V.); lindstranda@who.int (A.L.); 3 Duke-Robert J. Margolis, MD, Center for Health Policy, Washington, DC 20004, USA; sabine.sussman@duke.edu; 4 Duke Global Health Innovation Center, Durham, NC 27701, USA; katharine.olson@duke.edu (K.O.); rianna.cooke@duke.edu (R.C.); ku@duke.edu (K.U.); 5 Welch Medical Library, Johns Hopkins University, Baltimore, MD 21205, USA; 6 Direct Consulting and Logistics Limited, Abuja 901101, Federal Capital Territory, Nigeria; nwamaka.ezeanya@dclnigeria.com (N.E.); adefarrell@dclnigeria.com (A.A.A.)

**Keywords:** COVID-19 vaccines, service delivery, service integration, vaccination strategies

## Abstract

The COVID-19 vaccination is a crucial public health intervention for controlling the spread and severity of the SARS-CoV2 virus. COVID-19 vaccines have been developed in record time, but their deployment has varied across countries, owing to differences in health system capacity, demand for the vaccine, and purchasing power of countries. The aim of this rapid review is to summarize and synthesize experiences on COVID-19 vaccine service delivery and integration to inform future COVID-19 vaccination programming and contribute to the knowledge base for future pandemic management. A systematic search was conducted in PubMed, Scopus, and Global Index Medicus databases. Twenty-five studies were included in the analysis. Included studies spanned nine countries where COVID-19 vaccines were delivered through mass, mobile, and fixed-post vaccination service delivery models. There was limited evidence of integrating COVID-19 vaccines into routine services for pregnant women, people who inject drugs, and leveraging existing health programs to deliver COVID-19 vaccines to the general population. Common challenges reported were vaccine skepticism, lack of adequate health workers, and linguistic barriers to access. Partnerships with a variety of stakeholders and the involvement of volunteers were vital in overcoming barriers and contributed to the efficient functioning of COVID-19 vaccination programs.

## 1. Introduction

Vaccines are a critical tool to reduce severe disease and deaths from COVID-19. Unprecedented public and private investments and collaborations facilitated scientific breakthroughs that led to the development of several safe and effective COVID-19 vaccines in record time. Monumental efforts have been undertaken to turn vaccines into vaccinations, with more than 13 billion doses administered by end-January 2023 [1]. Of the world’s population, 63% had completed the COVID-19 vaccination primary series and 34% of the world’s population had received a booster dose as of December 2022 [1], and as a result, an estimated 20 million deaths have been averted [2]. However, COVID-19 vaccine delivery has been highly inequitable, with only 25% of the population in low-income countries having received at least one dose of the vaccine [1].

The inequities in vaccination rates have been driven by multiple supply- and demand-side factors throughout the pandemic. In the early stage of the pandemic, global vaccine supply was constrained, and many low-and-middle-income countries (LMICs) struggled to secure adequate vaccine supply [3,4,5,6]. While dose-sharing arrangements and vaccine diplomacy between countries occurred in some instances, predictable supply in LMICs was secured by early-2022 primarily through pooled procurement mechanisms such as COVAX and the African Vaccine Acquisition Task Team (AVATT). In contrast, supply in high-income countries was solely driven by bilateral deals and assured from early 2021.

As vaccine supply eventually increased in LMICs, additional challenges such as lack of operational budget to deliver the vaccines, low vaccine confidence due to myths, misconceptions and misinformation, and low-risk perception became dominant drivers of low vaccination rates [7,8,9]. Moreover, the shortage of healthcare workers due to high turnover rates exacerbated the challenges of the already strained healthcare infrastructure to deliver the incoming vaccines [10]. The task of COVID-19 prevention and control has been intricately complex with policymakers and public health leaders having to navigate multi-faceted and dynamic challenges as the pandemic evolved and evidence about the COVID-19 vaccines accrued. Though the future scale of COVID-19 vaccination for optimal population benefit is yet to be determined, current evidence on disease transmission and vaccine effectiveness indicates the need to increase coverage of the primary vaccine series followed by booster doses determined by the risk of severe disease and death in different sub-populations; the ‘highest priority’ group as recommended by WHO Strategic Advisory Group of Experts (SAGE) is older adults, health workers, and immunocompromised persons [11]. Targets for vaccinating the highest priority groups have not been met in several regions; as of 21 March 2023, a report of COVID-19 vaccination status in sub-Saharan Africa revealed that among 29 countries where data were available, 55% have vaccinated less than 50% of eligible older adults, 48% have vaccinated less than 50% of people with comorbidities, and 17% of countries have vaccinated less than 50% of health workers [12]. Therefore, going forward it is critical to review and reformulate strategies that would be required to increase vaccination rates among these highest priority target populations. 

The COVID-19 vaccine rollout experiences around the world have provided tremendous opportunities to learn from successes and challenges. While mass vaccination campaigns attempted to reach a large population in a short timeframe, the cost and impact on other services are important factors when considering the feasibility of campaign-mode delivery going forward. With the acute pandemic phase gradually receding, it is important to plan for the sustainability of COVID-19 vaccination to improve the efficiency and resiliency of vaccine delivery programs, curate innovative strategies to integrate vaccine delivery into other health services, and consolidate learnings for future pandemic management. This rapid review aims to synthesize experiences from the early phase of the COVID-19 vaccine rollout, which can be useful for informing the updates and changes to COVID-19 vaccine programming that are currently being discussed. This rapid review on service delivery and integration—one out of eight domains defined for a systematic review—aims to synthesize the experiences, learnings, and challenges in service delivery and integration of COVID-19 vaccination into immunization programs and primary healthcare services.

## 2. Materials and Methods

For this rapid review, the team, consisting of the WHO, the USAID-funded MOMENTUM Country and Global Leadership project, and the COVID GAP Collaborative at Duke University sought to balance the rigour of a systematic review with the timely delivery of a rapid review product based on the methodological guidelines of a rapid review as set forth by the Cochrane Collaboration [13]. We developed a conceptual framework to guide the review process that defined eight domains for inquiry: service delivery and integration, demand and uptake, supply and logistics, planning and coordination, political will and financing, health workforce, monitoring and evaluation, and adverse event monitoring and management. A search strategy using MeSH and Boolean operators was developed iteratively combining terms for COVID-19 vaccines and each of the above-mentioned domains (a detailed search strategy is provided in the Appendix A). The search was conducted in PubMed, Scopus, and Global Index Medicus databases, and inclusion/exclusion criteria were pre-ascertained. The inclusion criteria were:

- The eight domains mentioned above.
- Papers in the peer-reviewed literature.
- Papers where the reporting period was after the COVID-19 vaccine rollout (starting December 2020).

The exclusion criteria were:

- Lessons learned from other interventions and not COVID-19 vaccination.
- COVID-19 research protocols.
- COVID-19 vaccine effectiveness studies.
- Articles about the impact of the COVID-19 pandemic (except COVID-19 vaccine rollout) on routine immunization programs and/or other essential services.
- Papers that lack relevant data, i.e., data that can inform COVID vaccine rollout program decision making.

The screening process was managed on Covidence (https://www.covidence.org/, accessed on 4 May 2023, a platform for systematic review management. Two persons independently screened all titles and abstracts and tagged them by the domains. A third individual was nominated to resolve all conflicts. Papers that progressed to full-text screening were screened independently by 2 people, and the conflicts were resolved by the nominated arbitrator. The search was not restricted by language. Papers in French, Spanish, and Portuguese were screened by research team members proficient in those languages. In response to a request for rapid results from this review to inform a global compendium of country knowledge on COVID-19 vaccination hosted on the WHO TechNet site, a cut-off date of mid-July 2022 was set for full-text screening. Of the 86 full texts screened by the ascertained cut-off date, 25 papers were included in the analysis.

Quality appraisal tools for randomized control trials, qualitative, case-control, and cohort studies by the Critical Appraisals Skill Programme at the Oxford Centre for Triple Value Healthcare Ltd. were used to assess the papers for the validity of results, interpretation, and local relevance of the results [14]. Papers included in this analysis have versatile structures and do not necessarily lend themselves to the quality assessment tools which are designed strictly for research studies. Since the objective of this review is to summarize and synthesize learnings from the COVID-19 vaccine rollout, descriptive papers, commentaries, and research letters that spoke of strategies, successes, and challenges in vaccine implementation were been included. In addition, 68% of papers were assessed to meet all the quality criteria set forth in the respective tools, and while the rest partially fulfilled the criteria, they were included in the analysis for the value added by the important insights on service delivery and integration, that aligned with the goals of this paper (see Appendix A Table A1, Table A2, Table A3 and Table A4 for quality appraisal).

**Figure 1:**
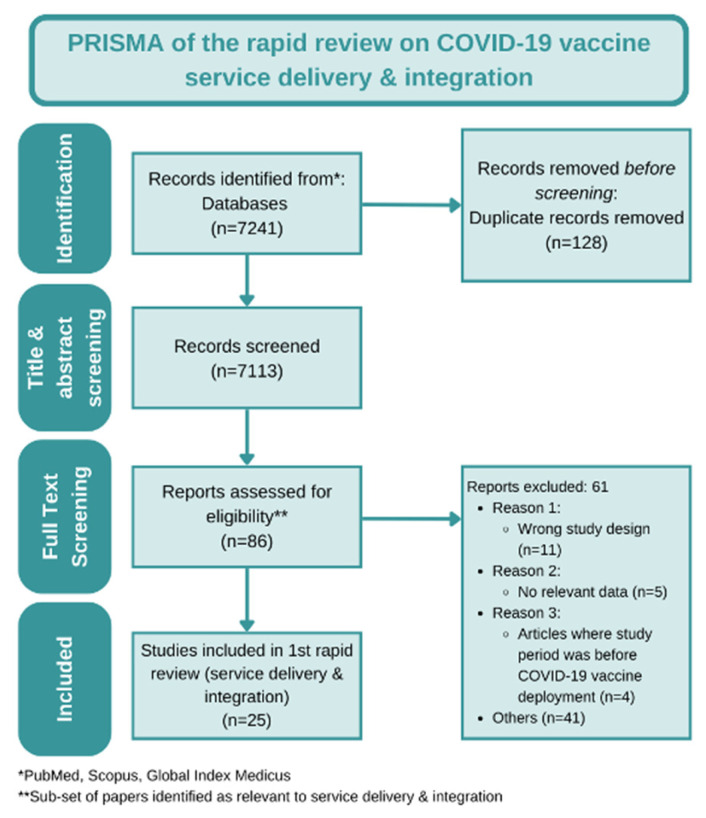
PRISMA diagram.

A data extraction tool was designed in Microsoft Excel to extract descriptive variables which were analyzed using qualitative methods on Dedoose, a CAQDAS platform for qualitative research. Ten sample papers were coded using inductive coding techniques to develop a preliminary code book. Two researchers reviewed, refined, and harmonized the codebook, which was then applied to all twenty-five papers (Figure 1—PRISMA diagram).

## 3. Results

The search yielded 7241 records of which 7113 were title and abstract screened (128 duplicates removed before screening). Search results included studies in English, Spanish, and Portuguese. Nine countries were represented in the 25 papers analyzed; the majority of the papers were from high-income countries (USA—13, Italy—2, Malta—2, Israel—2, Germany—1, England—1), 3 studies were from middle-income countries (South Africa—2, China—1), and 1 paper was from a low-income country (Sudan—1). Most papers reported short study durations (median duration = 2 months) and focused on early COVID-19 vaccine rollout experiences as 56% of the studies analyzed commenced within the first 4 months of initial COVID-19 vaccine rollout in December 2020. Target populations in studies were largely the general population (*n* = 16) and healthcare workers (*n* = 6), while a few focused on pregnant women (*n* = 2) and socially marginalized populations (injecting drug users) (*n* = 2).

There were three main study designs: case studies (*n* = 7) drawing data from program implementation reports (*n* = 4), health facility registers (*n* = 2) and secondary sources (*n* = 1); retrospective program evaluations (*n* = 8) drawing data from health facility records (*n* = 2), primary qualitative data (*n* = 2), internal and published reports (*n* = 2) and secondary sources (*n* = 1), 1 study did not report its data source; and cross-sectional studies (*n* = 4) drawing data from web-based surveys (*n* =1), state database (*n* = 1) and health facility registers (*n* = 2).

There was significant heterogeneity in the outcome indicators due to the eclectic mix of study designs and scope of documentation in the papers. Since this analysis included 25 papers only, the research team ascertained there was insufficient data on the various types of outcome indicators reported to include it in the summary analysis. Therefore, we synthesized insights from the papers into implications for three different vaccine delivery models—mass vaccination, mobile vaccination, and fixed-post vaccination—highlighting the differentiating factors of each model, and the enablers and barriers in service delivery from the perspective of each model. Table 1 summarizes the strategies used and lessons learnt in each of the papers included in the analysis. This rapid review is the first in a series of reviews on this topic, and the outcomes’ summary analysis will be included in subsequent publications where we will analyze a larger volume of papers.

### 3.1. Mass Vaccination Model

Eight papers from six countries (China—1, Israel—1, Italy—2, Malta—2, South Africa—1, USA—1) described vaccine delivery through the mass vaccination model. The model is typically characterized as high-volume, high-speed vaccination activities conducted in non-healthcare settings for rapid vaccine delivery during health emergencies.

Mass vaccination sites were observed to have five functional steps which were modified for different contexts. The first step is community mobilization and setting up appointment systems. We found evidence of both facility-initiated appointments [15,21] and voluntary registration for COVID-19 vaccination for qualifying groups prioritized in national or sub-national policy guidance [17,21,22]. There was evidence of flexibility in facility-initiated appointments for healthcare workers to accommodate the work schedules of those on healthcare duty [15]. In addition to scheduling options which were exclusively through digital tools, there was evidence of a mixed approach in Malta where invitation letters were sent via postal mail to some priority groups such as health workers, persons living in long-term care facilities, and people above 60 years of age in a staggered manner, and the rest were asked to book appointments via digital scheduling tools [21].

Registration and screening at the mass vaccination site was the second step in this process. Those seeking COVID-19 vaccines were registered on arrival at the site, screened for symptoms of COVID-19, and asked to provide informed consent for receiving the vaccine. Fischl et al. reported the use of multilingual COVID-19 vaccine information sheets and translators at the mass vaccination site who aided non-English speakers to overcome language barriers in providing informed consent for the vaccine [17].

The third step in this process is the history-taking of potential vaccinees. Medical histories, especially history of anaphylaxis, allergies, and other comorbidities, were documented. The fourth step is vaccination. Vaccination was conducted either in batches depending on the number of available vaccination stations [15], or one by one as people walked or drove in [17]. Signorelli et al. reported unifying the step of history taking and inoculation to reduce time wastage between the steps and increase convenience for elderly people and those with mobility restrictions [18].

The fifth and last step at mass vaccination sites is post-vaccination observation. Most studies documenting the mass vaccination process reported a 15 min mandatory post-vaccination wait for those with no prior history of allergies and a 30 min wait for those with a previous history of allergies [15,17,18,19]. Observation areas were equipped with back-up oxygen cylinders, crash carts, and stretchers for emergencies [16,17,20].

Challenges in implementing mass vaccination sites were experienced in both planning and coordination and securing adequate human resources to deliver COVID-19 vaccines at a large scale. Planning and coordination challenges included a lack of systems to categorize potential clients for vaccination by their risk level of contracting COVID-19 and the inability of digital appointment systems to optimize appointment slots in line with healthcare workers’ ability to get their shots [15]. Rosen et al. reported difficulty in securing appointments through digital scheduling tools during early rollout [22]. Reliance on clinicians to staff the mass vaccination sites without de-escalating or providing additional human resource coverage at their usual duty sites led to a compromise in non-COVID-19 related medical care [15]. Multi-sectoral partnerships have been consistently reported as means to overcome these challenges. Planning and coordination challenges were addressed through partnerships with information technology and public relations experts and organizations that provided scheduling support, site security, logistical management, and infection prevention and control [20,22]. Local universities extended volunteer support which filled in for logistical and clinical human resource support vital for the smooth running of the sites [18].

An anticipated challenge for COVID-19 vaccine delivery in mass vaccination mode that was well tackled was the transportation and handling of vaccines. Most of the reported vaccine types used had stringent cold storage needs and were meticulously dispensed from central cold stores to vaccination sites based on demand forecasting for the site [16], had regular quality checks of thawed vaccines [17], and employed innovative ideas such as using small insulated carry bags of maximum 20 doses and insulated boxes of the size of small pizzas for dispensing vaccines from central storage in small batches to avoid wastage [17,22].

### 3.2. Mobile Vaccination Model

Five papers from two countries (Germany—1, USA—4) reported COVID-19 vaccine delivery through mobile clinics. Mobile vaccination is an umbrella term to describe various initiatives to bring vaccination services closer to communities in need on a small scale. It typically targets rural areas, underserved minorities, and socially marginalized populations.

Delivery through the mobile vaccination model as observed in our analysis can be explained as a three-step process—community mobilization, appointment and registration, and inoculation. In contrast to the mass vaccination models where the emphasis for successful COVID-19 vaccine delivery was on efficient spatial arrangement and multi-sectoral partnerships to execute vaccination on a large scale, the focus of mobile vaccination models is on intensive and strategic community mobilization efforts to reach targeted populations through the mobile vaccine clinics. Locations for mobile vaccination sites were identified by zip code to target services to traditionally underserved areas that included vaccine sceptic populations [23], at locations where other popular health clinics were run [24], or at a place picked for its strategic importance of being familiar and family-friendly for the target population such as a church parking lot [25]. Mobile vaccination clinics had a combination of staff such as doctors, nurse practitioners, or pharmacists who could handle the vaccines and administer the doses [23,24,25]. Staff provided documentation and scheduling support for those who needed a second dose and covered the observations tents for post-vaccination monitoring and in some instances were supported by volunteers. Volunteers also had a variety of roles including registration of patients, helping with informed consent, and addressing questions. One paper reported using temperature-controlled coolers for temporary vaccine storage [23].

Some commonly reported strategies for mobilization were information, education, and communication (IEC) campaigns delivered through information channels and people who are trusted by the target population and those with an existing rapport with the community [25]. A faith summit was organized to mobilize the pastors’ support for the COVID-19 vaccine, deliver a comprehensive information session, and make the clergy’s support for the vaccine visible to the community [25]. Partnership with local organizations that have existing rapport with the community and a sound understanding of cultural context to support vaccination information dissemination was important for community mobilization [23,24]. A COVID-19 vaccine delivery program for people who inject drugs (PWID) attracted their existing client’s friends and family through the trust propagated with consistent engagement [24]. Since the vaccines had only received emergency use authorization and were yet to receive full regulatory authorization, instead of offering financial incentives for the COVID-19 vaccines, indirect financial incentives such as gift cards were provided for other services such as STI testing which were co-located with COVID-19 vaccines to attract more people to the clinic with the goal of increasing uptake for COVID-19 vaccination. Other non-financial incentives included offering drinks and snacks during post-vaccination observation to encourage COVID-19 vaccine uptake [24].

Religious and faith leaders played a crucial role in managing the registration and appointment lists for their communities which signalled their support and encouragement for COVID-19 vaccination [25]. Summer camps, high schools, federal housing facilities, elder care facilities, churches, and shelters were used as mobile vaccination clinic venues [23,32]. COVID-19 vaccine was delivered either as a standalone or an integrated service with needle exchange and HIV/STI testing services to PWID [23,24,25,26,32]. Translators, bilingual staff, and mobile applications for translation and facilitation of communication between provider and patient were important to complete the inoculation step [23,26].

Since mobile vaccination clinics typically target hard-to-reach populations who are marginalized due to a variety of factors including but not restricted to race, geographic location, conventionally unacceptable social practices such as drug injection, etc., an inherent challenge of this delivery model is vaccine scepticism due to historical distrust of the formal healthcare system, inequity, and opacity in vaccine allocation processes for marginalized groups, etc. [23,25]. The distrust was overcome by leveraging partnerships with local stakeholders who have an existing relationship with the community, and consistently engaging with the target population to comprehensively address their health needs [23,24]. Heidari et al. in their description of organizing mobile vaccine clinics for injecting drug users upheld the importance of serving every person who visited the clinic to propagate trust within the local community.

Cultural and language barriers created challenges in patient-provider communication and rapport which were addressed through counselling and using multilingual applications for facilitating communication between the patient and the provider [24,25,26].

### 3.3. Fixed-Post Vaccination Model

Thirteen papers from five countries (Israel—1, South Africa—1, Sudan—1, UK—1, USA—9) described COVID-19 vaccine delivery at fixed-post sites. While this model required the least spatial adaptation by virtue of being purpose-built for vaccination, community mobilization to encourage vaccine uptake was critical. There was an emphasis on conducting purposeful and contextually relevant IEC campaigns; Goga et al. described that the program addressed concerns about adverse events (AEFI) by employing a risk-benefit approach in the communication by acknowledging the rare risks of AEFI associated with COVID-19 vaccines and weighed the benefit of protection from severe disease inflicted consequences for unvaccinated people [39]. Two papers reiterated the importance of high-frequency and tailored messaging to address concerns specific to sub-population [32,33], and one-on-one counselling for real time-update on vaccine effectiveness for groups under-represented in clinical trials [29,31].

The appointment of vaccination champions and opinion leaders to advocate for the COVID-19 vaccine was also a commonly used strategy for mobilization. Medical and political leadership led conversations on the vaccine from the front, and key people from communities with diverse viewpoints were mobilized for consensus-building and dispelling myths, misconceptions, and rumours [27,29,32]. Another commonly used strategy for increasing vaccine demand and uptake was the provision of financial incentives such as gift cards and cash prizes for healthcare workers and the general population [35], and college scholarships for students [32]. Non-financial incentives such as t-shirts with motivational messaging for healthcare workers and snacks and drinks at clinics serving the general population were also offered to motivate vaccine uptake [27,28,32].

Although pre-registration appointments were common, some programs increased convenience for access by allowing walk-ins for those who missed appointments or were present at clinics without prior plans of getting vaccinated [29,39]. Multilingual appointment portals were also deployed for non-English speakers [32]. Adaptations to the consenting process included conducting the consent process online and over the phone for parents of students [33].

Challenges faced included vaccine scepticism due to distrust of the fast-paced vaccine development process [28], historical distrust in the formal healthcare system, fear of side effects, and misinformation [32,37]. A vaccination program in South Africa adopted the risk versus benefit approach in its communication strategy—acknowledging the rare chances of adverse vaccine reactions which are manageable but stressed on the higher probability of developing severe COVID-19 disease that can be life threatening and the key role of the vaccine in preventing it [39]. School-located vaccine programs in the US used multiple communication channels to disseminate vaccine-related information targeting parents, and in 2 districts in Colorado teachers incorporated vaccine science into lessons to debunk myths and misconceptions [32].

There were logistical constraints in vaccine storage and transportation due to inadequate infrastructure, an inequitable spread of health facilities that excludes certain sub-populations from accessing care, and the short shelf life of vaccines [34,37]. The lack of timely data on COVID-19 vaccine safety and efficacy for pregnant women posed challenges to uptake in this population [29]. As with mass vaccination and mobile vaccination models, one paper reported language barriers in understanding vaccine information sheets and consent forms for people who did not speak the common language [33]. A shortage of health workforce was also reported as a challenge in delivering COVID-19 vaccines [37]. The issue of health workforce shortage was addressed by building a volunteer force who in some instances such as nursing and pharmacy student volunteers even took on clinical duties of administering the vaccine [38]. Other approaches to addressing barriers to access such as lack of transportation or technological know-how were identifying people facing these barriers and subsequently removing the barriers by arranging transportation to the vaccine clinic, providing internet booking for those who needed it, and allowing walk-ins [32].

Partnerships with the private sector for the running of fixed-post vaccination sites improved vaccine sourcing, staffing, and maintenance of data systems, and partnerships with civil society organizations helped mobilize human resource support for vaccine distribution and operations [32,33].

### 3.4. Evidence of Integration of COVID-19 Vaccines with Other Health Services

Four papers from three countries (Israel—1, UK—1, USA—2) described efforts to integrate the COVID-19 vaccine with health services which are either routinely accessed by certain sub-populations such as people who inject drugs and pregnant women or services for the general population at large.

The first type of integration identified in the analysis was the co-location of COVID-19 vaccine clinics and needle exchange programs and HIV/STI testing for people who inject drugs, and 20-week antenatal check-ups for pregnant women, respectively [24,29]. Heidari et al. describe a city-level program in the US where two vans which served as mobile vaccine clinics and needle exchange and STI testing clinics were co-located in selected areas on certain days, and people who regularly visited the needle exchange clinics were counselled by the clinic staff on the benefits and importance of receiving the COVID-19 vaccine. Those who were interested in getting the vaccine were linked to the vaccine clinic where they received the vaccine. The syringes service program staff routinely walked their clients to clinicians on site to ensure any questions and doubts are addressed [24].

Similarly, Cater et al. described the piloting of a dedicated, fast-track COVID-19 vaccine clinic in a vaccine hub serving the general population at a hospital site. Pregnant women who visited the antenatal clinic for their 20-week check-up and scan were targeted for COVID-19 vaccines. Invitation letters for COVID-19 vaccination were sent to women due for their 20-week antenatal check-up. Healthcare staff at the antenatal clinic counselled the pregnant women on the day of the 20-week appointment about the importance of COVID-19 vaccines and their availability at the hospital site. They made verbal offers for same-day appointments. Women who consented to COVID-19 vaccines were referred to the dedicated, fast-track COVID-19 vaccine clinic for pregnant women and were offered the vaccine on the same visit. A colour-coded fast-track card was issued to the women who consented to the vaccine which allowed operational streamlining at the vaccine hub. Obstetricians, gynaecologists, and leadership of these departments made themselves available at the vaccination site to answer questions and address doubts.

In both these examples of co-location of services, the counselling, and referral by healthcare providers who regularly interact with these populations and are trusted were crucial in helping people decide on accepting the vaccine. The co-location of services also reduced the burdens of additional costs for transportation and child care [29].

The second type of integration as seen in this analysis is the co-delivery of COVID-19 vaccines with influenza vaccines in a school-located vaccine clinic which targeted the general population through students, teachers, and other school affiliates [32]. The school-located vaccine clinic program in a district in the USA appointed a school nurse to deliver COVID-19 and influenza vaccines. A teacher leveraged this mobile vaccine clinic service as an opportunity to educate students on the safety and necessity of vaccines and received her influenza shot during class in an effort to mobilize positive sentiments for the vaccine.

The third type of integration was the co-use of existing infrastructure for blood donation camps and COVID-19 vaccination campaigns in Israel [36]. The infrastructure required for blood donation operations was reused for COVID-19 vaccination which included proper sites for the assessment of individuals’ eligibility before vaccination, the actual procedure, and the post-vaccination observation period.

Challenges in integration were discussed only in the first type (co-location of services) of service integration mentioned above. Cater et al. described challenges with vaccine scepticism due to insufficient vaccine safety and efficacy data for pregnant women. Up until April 2021, pregnant women in the UK were advised not to get vaccinated and by the time this pilot was implemented in June 2021, the guidelines were changed, and it recommended the COVID-19 vaccine for pregnant women. This seemingly swift change in guidelines created doubts for some and contributed to scepticism [29]. Vaccine counselling was primarily delivered through midwives in the antenatal clinic; there were reports of instances where the midwife delivered the vaccine information required by the protocol but also communicated personal hesitation about the vaccine for pregnant women, thereby influencing scepticism among pregnant women. The other challenge mentioned was the disruption to clinical services offered by the needle exchange program due to lockdowns in the US [24]. Heidari et al. described the move to a tele-medicine clinic to continue some services such as access to medications for opioid use disorder but it was not clear how the program coped with providing in-person services such as HIV/STI testing and COVID-19 vaccines.

## 4. Discussion

### 4.1. Key Learnings on COVID-19 Vaccine Service Delivery Models and Integration

The primary goal of this rapid review was to learn from experiences, successes, and challenges of delivering COVID-19 vaccines with a special focus on learnings in service delivery models for high priority-use populations—namely, elderly people, people with comorbidities and immunocompromised, health workers, pregnant women, and socially marginalized populations. Most studies focused on describing COVID-19 vaccination targeting the general population, followed by some on vaccination of healthcare workers, and a couple of papers documented experiences for pregnant women, and socially marginalized groups. More than half the papers described experiences and learnings from the early COVID-19 vaccine rollout when policies largely prioritized healthcare and other frontline workers due to the high-risk nature of their jobs and the limited global COVID-19 vaccine supply [40,41,42]. Nearly one-third of the papers described mass vaccination models, the go-to delivery model to interrupt a rapidly spreading pandemic as it can achieve fast vaccine coverage in a shorter period compared to other models. The dominance of high-income countries in this review testifies that these countries were able to successfully implement mass vaccination models early in the vaccine rollout period because of the availability of sufficient vaccine supply. The early experience of LMICs did not start appearing until much later due to the lag in publication and probably why there is a dearth of papers from LMIC settings during the period this rapid review covered. Even so, focusing on the early experience helps us contextualize the pandemic response at that time. The response has since evolved as we progressed and learnt along the way. However, the learnings from this rapid review will contribute to scholastic memory of the strategies to adopt, the challenges programs may face, and the enablers that can power an early pandemic response when infection rates are high and vaccine availability is limited.

Mass vaccination models followed a set of standardized steps starting from appointment booking to post-vaccination observation. Challenges in mass vaccination models included planning and coordination of these massive operations, securing adequate workforces for all necessary functions, diversion of health workforce from other health services for mass vaccination duties, and efficient implementation of appointment systems [15,18,20,22]. The anticipated challenge of vaccine storage and handling was shown to be tackled through meticulous demand forecasting and vaccine dispensation from cold stores to vaccination sites, and innovative solutions that enabled the transportation of vaccines in small batches to avoid wastage [16,17,22]. Partnerships were the key enabler that helped overcome a lot of the above-stated challenges in mass vaccination sites [18,20,22].

Evidence on the mass vaccination model primarily described the spatial organization of the vaccination sites and strongly emphasized the need for multi-sectoral partnerships and multi-disciplinary teams owing to the massive logistical and operational efforts necessary for the efficient delivery of vaccines through this model [15,16,17,18,19,20]. The time-sensitive nature of mass vaccination efforts for COVID-19 challenged planners not only to deliver at scale but also at a rapid pace, and partnerships with non-healthcare and private sector stakeholders have been critical in implementing these. Private sector stakeholders included local pharmacies, universities, and private healthcare companies who provided technical support for setting up and maintenance of the vaccination sites [18], logistical planning and coordination for local cold chain management, storage, and transportation of vaccines, and supplemented human resources at vaccination sites [15]. These also included interdisciplinary partnerships between the medical and non-medical professions of physicians, nurses [15], public health professionals [20], logisticians, pharmacists [15], and architects [18]. Private sector partnerships have also been crucial for service delivery through mobile vaccination and fixed-post vaccination models where private sector stakeholders played similar roles in personnel supply, planning, and logistics coordination [32,33,39], and registration and billing support [33]. These linkages formed with private sector partners are assets and should be retained, further nurtured, and utilized to supplement skills in which the private sector has a comparative advantage, to overcome operational challenges for healthcare delivery. Developing sustained relationships with these partners will also better position public health programs in pandemic preparedness to swiftly respond to future global health crises.

Implementation of mobile vaccination models can be summarized as a 3-step process—community mobilization, appointment, and registration. Locations for mobile vaccination sites were strategically chosen to reach target populations in places that were convenient and acceptable to the people. Papers described a host of strategies such as the implementation of IEC campaigns through trusted messengers, engagement of respected local leaders and organizations in IEC efforts, leveraging relationships with the target population to reach families and friends beyond the core target group, and providing incentives to inspire interest in vaccines for community mobilization efforts [23,24,25]. Religious and faith leaders were tasked to manage appointment and registration systems. Challenges in this model were vaccine scepticism due to historical distrust of the healthcare systems, cultural and linguistic barriers, and intra-country inequity in vaccine allocation processes for marginalized groups [23,25]. Enablers included community mobilization efforts and partnerships with local stakeholders to overcome some of the above-mentioned barriers [23,24].

The studies on fixed-post vaccination did not focus much on the organization of the model, since it is purpose-built for vaccination and other healthcare activities. These papers emphasized community mobilization to encourage vaccine uptake. Strategies used in this model were the appointment of vaccine champions and opinion leaders, provision of financial and non-financial incentives, and implementation of tailored, IEC campaigns [27,29,33,35]. There was some discussion on managing and adapting appointment systems by allowing walk-ins and easing the consenting processes through online and phone consent options. Challenges highlighted in this model were vaccine scepticism, logistical constraints of vaccine storage and transportation, linguistic and technological barriers to access, and shortage of health workforce [28,32,34,37]. Enablers in this model that overcame some of these challenges were community mobilization efforts and partnerships [38].

Based on the papers analyzed in this review, such as the mass vaccination model, although partnerships in mobile vaccination and fixed-post vaccination model of delivery were crucial [23,24,25,27,28,29,34,35,39], there was a greater focus on community mobilization to encourage people to come to the clinic. A striking similarity between the strategies used in these two models of delivery was the importance of consistent, prolonged engagement with the target population prior to offering COVID-19 vaccines [24,29,31]. Programs that used healthcare teams and structures that were trusted and had an existing rapport with the community and added the COVID-19 vaccine as an added service to the existing continuum of services reported more positive experiences in generating demand and achieving higher vaccination coverage. Given the rumours and COVID-19 vaccine scepticism, using trusted community leaders and vaccine champions to deliver messages on the safety and effectiveness of COVID-19 vaccines has proven useful to build vaccine confidence and generate demand. COVID-19 vaccine delivery programs and health programs, in general, should adopt consistent community engagement as a core principle to leverage the trust that has been built during COVID-19 vaccination efforts and develop longer-term community engagement initiatives through which other essential services can be delivered regularly and specific services when future potential pandemics hit.

In addition to the three-vaccine delivery models we discuss here, there is evidence of other delivery models such as home-based vaccine delivery which were implemented to reach vulnerable or hard-to-reach populations [43]. As demand pressure eased and vaccine supply stabilized, vaccine delivery efforts evolved and more had to be done to take vaccines to the people. Discussion on other evolving delivery models that have been implemented is beyond the scope of this paper since our rapid search did not yield those results, but we hope future search updates and iterations to this analysis will allow for a deeper discussion on alternate vaccine delivery models which are critical to close the coverage gaps.

This review yields a summary of strategies implemented across the 3 models. Despite some commonalities in strategies between models and between studies of the same model, every program captured in this review is unique in implementing a set of strategies tailored to its programmatic needs. For example, a program built for injecting drug users prioritized the co-location of STI/HIV testing services with COVID-19 vaccines and provided incentives to improve uptake [24]. However, a program for pregnant women which also co-located the COVID-19 vaccine with antenatal services prioritized one-on-one counselling services to address doubts on vaccine safety and efficacy for pregnant women since at the time there was a dearth of evidence for this target group [29].

Through a combination of vaccine delivery strategies, programs have achieved impressive vaccination goals on a massive scale in a short timeframe, but this has come at the cost of the diversion of resources from other health services and burnout among healthcare workers [44,45]. The current evidence on the waning of natural and vaccine immunity provides reason to believe that periodic booster doses for vulnerable populations will be needed. We see the response shifting from a disruptive vertical model to a more sustainable integrated service with primary healthcare and other health services [46]. There is an ongoing need to evaluate, plan and invest in re-designing strategies for the sustainability of future COVID-19 vaccination.

Until now most immunization programs in LMICs have focused on discrete life stages such as childhood and adolescents. COVID-19 vaccine delivery has demonstrated in a quick span of time the feasibility and learnings for vaccinating the adult population, and the nuts and bolts of expanding universal access to vaccines for vulnerable groups who had previously not had access to vaccination [47]. Current research should prioritize documenting lessons on vaccine delivery for adult vaccination in LMICs and expanding access to vulnerable populations to inform future efforts of an inclusive life-course approach to immunization.

### 4.2. Adaptations to Strategy

Findings in this rapid review are from COVID-19 vaccine delivery in the acute phase of the pandemic when the supply pipeline was still being built and vaccine demand was high. We have since moved towards a phase where there is sufficient COVID-19 vaccine supply, but demand is lower in some places [3,48]. This calls for adaptation to vaccine delivery and demand generation strategies to reach unvaccinated people and define strategies to periodically reach those in need of additional booster doses. A proportion of papers reported using pre-visit appointment systems, and some highlighted the benefits of allowing walk-ins [15,17,32,33,34]. Now with sufficient supply, it is encouraging to see appointment systems tweaked to encourage walk-ins and incorporated into the messaging strategy to remove technological and linguistic barriers to access [32,33,39]. This adaptation is especially crucial for LMICs since internet connectivity is largely skewed towards people in urban centres. In addition, it is important to establish new entry points for high-risk group vaccination as part of primary healthcare or other health services reaching these risk groups that may integrate COVID-19 vaccination into their regular services. Our review found that the predominant framing of messaging was the risk–benefit approach. Given the slump in COVID-19 vaccine demand, a shift in messaging strategy from one that addresses falling risk perception to a strategy that addresses the falling risk perception of COVID-19 with a focus on those at higher risk of severe disease and mortality will be important to fill existing demand gaps.

In this review, the lack of COVID-19 vaccine acceptance was frequently reported as a challenge in the rollout. Commonly reported reasons for hesitancy in this review were distrust of the formal healthcare system and concerns about product safety and its quick pace of development. Irrespective if a country is high- or low-and-middle-income, this calls for imparting tailored, frequent, evidence-based messages to address concerns specific to different sub-populations. We found evidence of the power of leveraging community relationships, building rapport through consistent investment and work in the local communities, and partnering with community vaccine champions to bridge the trust and knowledge gaps [22,27,28,32,33]. The opportunity to discuss questions and concerns about the COVID-19 vaccine with a healthcare provider or someone with demonstrated knowledge of healthcare such as pharmacists also proved beneficial for generating trust in and demand for the vaccine [25,29]. Programs should invest in the inter-personal communication of health workers and other relevant providers through more one-to-one outreaches, cultivate vaccine champions from within the community whose opinion is trusted, and engage varied voices in the conversation with the goal of consensus-building and not just convincing.

### 4.3. Limitations of the Study

This paper adheres to the methodology of a rapid review as recommended by the Cochrane group [13]. Since one of the goals of this rapid review was to generate rapid learnings to inform ongoing conversations on COVID-19 programming, the literature search was limited to peer-reviewed sources to ensure the feasibility of the project in the given timeline. The learnings from this paper should be situated in the context of the early vaccine rollout period which was characterized by high infection rates and limited global vaccine supply. Many challenges and learnings from that phase may not be relevant in refining strategies for the current context of the COVID-19 vaccine rollout and its future. More importantly, the successes and challenges discussed in this paper are overwhelmingly representative of the experience in high-income countries. There is a dearth of insight in this review on the COVID-19 vaccine rollout in LMICs and the strategies used in resource-limited settings to overcome the challenges of vaccination. Development and academic partners need to invest and support LMICs to generate, synthesize, and document evidence on the COVID-19 vaccination implementation program in these settings both for informing programming for adult vaccination and for future pandemic preparedness. The research team recognizes the dynamic nature of the pandemic and the fast pace at which evidence is being generated. The search for this review will need to be updated to include papers from regions that are currently not well-represented in this analysis.

## 5. Conclusions

In the early part of the pandemic high-income countries adopted three service delivery models for COVID-19 vaccine delivery—mass, mobile, and fixed-post vaccination models. Some strategies to improve vaccine delivery used across the models were the appointment of vaccine champions, providing different types of incentives, moving vaccination closer to communities and in spaces where communities frequently visit, introducing flexibility in appointment scheduling, rolling out targeted communication campaigns, and establishing partnerships with a wide variety of stakeholders.

For health program strategists and planners, it is important to note that while there were three dominant models in this review there was no one-size fits all solution to improving and sustaining vaccine uptake. As highlighted in the discussion, it is important to tailor strategies to the context and maturity of the program.

Planners should also note that the usefulness of strategies is context- and time-dependent. As we move through the various stages of the pandemic, the severity of the virus evolves, and the balance between demand and supply shifts from being supply constrained to supply-sufficient, these strategies will need to be tweaked to serve the prevalent programmatic needs.

As a closing thought, the pandemic response is a constant learning process. Program strategists and planners must constantly learn from ongoing experiences and be swift in implementing tweaks to the response.

## Figures and Tables

**Table 1:** Strategies used for COVID-19 vaccination and lessons learnt.

Author (First)	Country	Reporting Period	Target Population	Strategies Used	Recommendations/Learnings
Mass Vaccination Model					
Reddy et al. [15]	South Africa	17 February to 26 February 2021	Healthcare workers	Pilot program helped identify bottlenecks before full-scale rollout. Pre-assigned appointment system with flexibility to reschedule minimized staff-time wastage. Sizeable coordination team ensured smooth flow of mass vaccination campaign.	Ensure that vaccinators and handlers are trained, supported, and rested. Strengthen the occupational health and safety team. Create clear communication channels between all stakeholders. Have a pre-registration system and a back-up list to avoid wastage of vaccine doses. Encourage visible involvement from hospital management. Conduct a ‘dry-run’ of the vaccination process before starting the rollout.
Brambilla et al. [16]	Italy	21 January to 21 February 2021	General population	Interactive tool developed to forecast equipment, space, cost, and operator needs.	None reported by authors.
Fischl et al. [17]	USA	24 February to 6 April 2021	General population	Stepwise vaccine dispensation for small quantity doses in insulated bags based on queue length. Multilingual IEC materials and consent forms.	Live estimation of vaccine doses through quick and simple methods reduced wastage of vaccines.
Signorelli et al. [18]	Italy	20 April to 4 May 2021	General population	Intensive training for all personnel categories. Multi-disciplinary teams to complete all activities in one step to avoid time wastage.	Reducing the number of steps in the vaccination workflow reduces the wait time and makes it easier for elderly people.
Grech et al. [19]	Malta	27 December 2020 to 15 April 2021	General population	Accurate demand projection and early vaccine orders for stable vaccine pipeline. Coordination and partnership with multiple stakeholders for planning.	Develop contingency plans for unpredictable vaccine supply.
Jin et al. [20]	China	March to April 2021	General population	Trial and error method used to find optimum composition of immunization team for maximum efficiency. Vaccination site located at easily accessible location.	Temporary COVID-19 vaccination sites can be adapted for vaccination sites of tropical diseases which are endemic in the region.
Cuschieri et al. [21]	Malta	December 2020 to 9 August 2021	General population	Incremental increase in storage capacity to avoid stock-out. Variety of appointment systems to suit the need of populations.	Multiple systems for vaccination appointments implemented in staggered manner to reach high-risk groups first.
Rosen et al. [22]	Israel	14 December to 30 December 2020	General population	Innovative vaccine distributions in small batches helped remote sites. Multi-pronged appointment scheduling system accommodated different needs. IEC campaigns delivered through traditional and digital media and active approach to tracking and addressing anti-vax messages. Vaccine champions recruited from the community (key religious leaders).	Splitting up of large vaccine batches supply into smaller lots can enhance the number and geography of vaccination sites.
Mobile Vaccination Model					
Alcendor et al. [23]	USA	March to September 2021	General population—underserved minorities	Translators and bilingual providers at vaccination sites. Multilingual IEC campaigns in partnership with community-based organizations.	Partnership with community-based organizations increased the volunteer base and fostered trust. Partnering with faith-based organization provided in-roads into skeptic communities.
Heidari et al. [24]	USA	Starting Spring 2021	Injecting drug users	COVID-19 vaccine was offered as an add-on to regular services. One-on-one counselling support. Non-financial incentives provided.	Provide comprehensive package of services of interest to the target population.
Abdul—Mutakabbir et al. [25]	USA	21 January to 20 February 2021	General population	Recruited domain experts from the community to conduct IEC sessions. Community-based domain expert tasked with high-visibility role to garner community’s trust. COVID-19 faith summit was organized to mobilize support of religious leaders.	Assign visible roles for demand generation and vaccine administration to experts who belong to the target community.
Noack et al. [26]	Germany	Not specified	General population	Multilingual mobile application to facilitate communication between providers and patients. Incorporated sign language by means of video output for people with hearing impairment.	Mobile applications should work on culturally sensitive communication for ethnic minority patients in addition to addressing the linguist barriers to access.
Fixed-Post Vaccination Model					
Berry et al. [27]	USA	4 February to 2 March 2021	Healthcare workers and elderly people	Information, education, communication (IEC) delivered in-person and through digital platforms. Enforcement of declination forms for vaccine sceptics. Frontline staff as vaccine champions. Goal setting at facility-level. In-kind incentives.	Facilities should employ a combination of strategies to improve coverage.
Berry et al. [28]	USA	December 2020 to March 2021	Healthcare workers	Virtual townhall with leaders of diverse opinions. Distribution of audio-visual education materials. In-kind incentives.	Critical to train opinion leaders on empathetic and confident communication techniques.
Cater et al. [29]	England	28 June to 30 September 2021	Pregnant women	One-on-one counselling support. IEC activities by maternity department leaders on real-world data, through digital media, and in antenatal clinics. Walk-ins for vaccination accommodated.	Transparent exchange of evidence builds trust in COVID-19 vaccines for pregnant women. Visible involvement of leadership and maternity care teams in vaccine promotion is crucial for confidence building. Strategic positioning of vaccination clinics closer to pregnant women reduced burden of travel and improved uptake and equity in access. Continuity of care teams in delivering antenatal care and COVID-19 vaccination enabled to reach hard-to-reach populations.
Martinez et al. [30]	USA	Inception of COVID-19 vaccination to 20 September 2021	General population	IEC using culturally tailored messages. Convenient vaccination locations. Comprehensive test, trace, treat program built trust among the community and benefitted vaccination efforts. Community health worker led programs funded for IEC, contact tracing, and appointment scheduling.	Inter-sectoral and inter-professional partnership was critical for vaccine rollout. Transportation support removed a barrier to vaccine access. Strong advocacy for inclusion of undocumented migrants improved coverage in border areas.
Hirshberg et al. [31]	USA	27 April to 20 May 2021	Pregnant women	COVID-19 vaccines available at antenatal clinics. One-on-one counselling on COVID-19 vaccines.	In addition to ensuring vaccine availability, implementing strategies to address vaccine scepticism is critical
Behrmann et al. [32] *	USA	August 2019 to late summer 2021	School students and general population	IEC campaigns delivered targeted messages for students and their families on safe return to school, privacy of immigration details, etc. Vaccination mandates for students. Amended school curriculum with vaccine education. Appointment of vaccine champions. Financial and non-financial incentives. Multilingual appointment websites and consent forms. Walk-in clinics.	Parents should be targeted with vaccine awareness campaigns to increase coverage among students. Need for catch-up campaign for other antigens as resources were diverted from other vaccines for school-age children. School-based clinics could integrate annual flu and COVID-19 vaccines.
Behrmann et al. [33]	USA	Not specified	School students and general population	IEC campaign targeted families of students. Appointment of vaccine champions from the community. Walk-ins allowed. Used telephone and electronic platforms for parental consent.	Employ engaging methods for demand generation over passive methods. Simplify the consent forms for ease of reading and understanding.
Andrade et al. [34]	USA	15 December to 29 December 2020	Healthcare workers	The IEC campaign was designed to address HCWs’ vaccine apprehension. Medical students incorporated into medical and administrative roles. Regular reminders sent to sign up.	Greater involvement of pharmacists in vaccination efforts can optimize vaccination efforts, especially during a health emergency.
Fareed et al. [35]	USA	23 December 2020 to 31 January 2021	General population	IEC campaign led by facility leadership. Financial incentives provided.	None reported by authors.
Jaffe et al. [36]	Israel	Not specified	General population	Offer COVID-19 vaccines at blood donation drives.	Due to similar operational requirements, blood donation drive sites can be adapted to vaccination sites.
Mohamed et al. [37]	Sudan	Not specified	General population	Public awareness campaign organized in partnership with multiple stakeholders and had a wide reach including internally displaced populations.	In settings with limited vaccine availability, residents should be categorized by priority and vulnerability. Need for health infrastructure investment for better vaccine storage and transportation. Establish security plans to prevent unlawful access to vaccine storage facilities.
Sanchez et al. [38]	USA	December 2020 to April 2021	Healthcare workers	Appointments issued through invitation with flexibility for rescheduling. Open layout for vaccination clinic to enable easy patient flow and oversight.	For university-based clinics, early involvement of students is useful for efficiency and to avoid wastage. Recruiting volunteers from the university enabled rapid scale up of operations.
Goga et al. [39]	South Africa	17 February to 26 May 2021	Healthcare workers	IEC strategy used a cost-benefit approach to messaging for addressing adverse event concerns. Walk-ins for people who had missed appointment notifications.	Vaccination sites should be expanded to convenient locations. Partnership with local religious and community leaders is essential.

* Paper also provides evidence on the mobile vaccination model.

## Data Availability

Not applicable.

## Appendix A

**Table A1:** Quality appraisal of qualitative studies.

	Section A: Are the Results Valid?						Section B: What Are the Results?			Section C: Will the Results Help Locally?
Paper	1. Was There a Clear Statement of the Aims of the Research?	2. Is a Qualitative Methodology Appropriate?	3. Was the Research Design Appropriate to Address the Aims of the Research?	4. Was the Recruitment Strategy Appropriate to the Aims of the Research?	5. Were the Data Collected in a Way That Addressed the Research Issue?	6. Has the Relationship between Researcher and Participants Been Adequately Considered?	7. Have Ethical Issues Been Taken into Consideration?	8. Was the Data Analysis Sufficiently Rigorous?	9. Is there a Clear Statement of Findings?	10. How Valuable Is the Research?
Cater	Yes	Yes	Yes	Yes	Yes	Yes	Yes	Yes	Yes	Further research on providing pregnant people with guidance and support to get the COVID-19 vaccine
Cuschieri	Yes	Yes	Yes	Yes	Yes	Yes	Yes	Yes	Yes	Examines Malta’s vaccination strategies (as a successful case study) to inform other countries how to implement successful vaccination techniques
Grech	Yes	Yes	Yes	Yes	Cannot tell	Yes	Yes	Cannot tell	Yes	Examines Malta’s mass vaccination campaign by touring a facility
Martinez	Yes	Yes	Yes	Yes	Yes	Yes	Yes	Yes	Yes	Studies vaccine equity among Hispanic border communities in California to exemplify how equitable access is possible
Behrmann	Yes	Yes	Yes	Yes	Yes	Yes	Yes	Yes	Yes	Provides a background for school-located vaccination clinics for planning and implementation
Reddy	Yes	Yes	Yes	Yes	Yes	Yes	Yes	Yes	Yes	Presents lessons and challenges from mass vaccination campaign from the largest hospital in Africa
Behrmann	Yes	Yes	Yes	Yes	Yes	Yes	Yes	Yes	Yes	Sharing of experiences for insights on school-located vaccination clinics and implementation
Alecendor	Yes	Yes	Yes	Yes	Yes	Yes	Yes	Yes	Yes	Provides insights on the implications of increasing vaccine uptake with the use of a mobile vaccination clinic
Andrade	Yes	Yes	Yes	Yes	Yes	Yes	Yes	Yes	Yes	Explains the implementation process of a pharmacist-led vaccination clinic in a hospital setting
Brambilla	Yes	Yes	Yes	Yes	Yes	Yes	Yes	Yes	Yes	Shares insights into the process of development of a scalable model for mass vaccination clinics
Fareed	Yes	Yes	Yes	Yes	Yes	Yes	Yes	Yes	Yes	Explains the implementation of a COVID-19 vaccination plan in substance use disorder residential settings
Fischl	Yes	Yes	Yes	Yes	Yes	Cannot tell	Yes	Cannot tell	Yes	Presents lessons learned and provides insight for mass medical operations
Jaffe	Yes	Yes	Cannot tell	Yes	Cannot tell	Cannot tell	Cannot tell	Cannot tell	Yes	Presents the experience of using blood services platforms to facilitate COVID-19 vaccination in Israel
Heidari	Yes	Yes	Yes	Yes	Yes	Yes	Yes	Yes	Yes	Presented lessons learned from integrating COVID-19 vaccination with syringe service and other communicable disease testing programs
Mohamed	Yes	Yes	Yes	Yes	Cannot tell	Yes	Yes	Cannot tell	Yes	Explored challenges to COVID-19 vaccination in the Darfur region of Sudan
Sanchez	Yes	Yes	Yes	Yes	Yes	Yes	Yes	Yes	Yes	Presents lessons learned and challenges of developing a COVID-19 vaccination program at a health sciences university
Rosen	Yes	Yes	Yes	Yes	Yes	Yes	Yes	Yes	Yes	Examines factors that enabled Israel to rapidly rollout COVID-19 vaccinations
Signorelli	Yes	Yes	Yes	Yes	Yes	Yes	Yes	Yes	Yes	Provides insights on the planning, implementation, and evaluation of a mass vaccination model utilizing vaccine islands
Goga	Yes	No	Yes	Yes	Yes	Yes	Yes	Yes	Yes	Presents lessons learned to strengthen national and global vaccination strategies, based on a vaccine implementation study of healthcare workers in South Africa
Noack	Yes	Yes	Yes	Yes	Yes	Yes	Yes	Yes	Yes	Discusses development of a multilingual app to provide information regarding COVID-19 vaccination to promote vaccines to groups with limited language proficiency
Jin	Yes	Yes	Yes	Yes	Cannot tell	Yes	Yes	Cannot tell	Yes	Examines the creation of a temporary COVID-19 vaccination clinic
Abdul-Mutakabbir	Yes	Yes	Yes	Yes	Yes	Yes	Yes	Yes	Yes	Presents strategies for effectively reaching the Black community with COVID-19 vaccinations

**Table A2:** Quality appraisal of case-control studies.

			Section A: Are the Results of the Trial Valid?			Section B: What Are the Results?			Section C: Will the Results Help Locally?			
Paper	1. Did the study address a clearly focused issue?	2. Did the authors use an appropriate method to answer their question?	3. Were the cases recruited in an acceptable way?	4. Were the controls selected in an acceptable way?	5. Was the exposure accurately measured to minimize bias?	6. (a) Aside from the experimental exposure, were the groups treated equally?	6. (b) Have the authors taken account of the potential confounding factors in the design and/or in their analysis?	7. How large was the treatment effect?	8. How precise was the estimate of the treatment effect?	9. Do you believe the results?	10. Can the results be applied to the local population?	11. Do the results of this study fit with other available evidence?
Berry	Yes	Yes	Yes	Yes	Yes	Yes	Yes	Multiple AORs reported. Some significant, others not	4 significant results. For those, ranges were (1.0, 1.3), (1.1, 6.6), (1.2, 8.9), (1.5, 19.6)	Yes	Yes	Cannot tell

**Table A3:** Quality appraisal of cohort studies.

		Section A: Are the Results of the Study Valid?			Section B: What Are the Results?					Section C: Will the Results Help Locally?				
Paper	1. Did the study address a clearly focused issue?	2. Was the cohort recruited in an acceptable way?	3. Was the exposure accurately measured to minimise bias?	4. Was the outcome accurately measured to minimise bias?	5. (a) Have the authors identified all important confounding factors?	5. (b) Have they taken account of the confounding factors in the design and/or analysis?	6. (a) Was the follow up of subjects complete enough?	6. (b) Was the follow up of subjects long enough?	7. What are the results of this study?	8. How precise are the results?	9. Do you believe the results?	10. Can the results be applied to the local population?	11. Do the results of this study fit with other available evidence?	12. What are the implications of this study for practice?
Hirshberg	Yes	Yes	Yes	Yes	No	No	Yes	Cannot tell	3% vs. 11% vaccine uptake before and after the exposure	Not significant (*p* = 0.22)	No	Cannot tell	Cannot tell	Can inform design of future research projects

**Table A4:** Quality appraisal of randomized controlled trial.

Section A: Is the Basic Study Design Valid for a Randomized Controlled Trial?			Section B: Was the Study Methodologically Sound?			Section C: What Are the Results?				Section D: Will the Results Help Locally?			
Paper	1. Did the study address a clearly focused research question?	2. Was the assignment of participants to interventions randomized?	3. Were all participants who entered the study accounted for at its conclusion?	4. (a) Were the participants ‘blind’ to intervention they were given?	4. (b) Were the investigators ‘blind’ to the intervention they were giving to participants?	4. (c) Were the people assessing/analyzing outcome/s ‘blinded’?	5. Were the study groups similar at the start of the randomized controlled trial?	6. Apart from the experimental intervention, did each study group receive the same level of care (that is, were they treated equally)?	7. Were the effects of intervention reported comprehensively?	8. Was the precision of the estimate of the intervention or treatment effect reported?	9. Do the benefits of the experimental intervention outweigh the harms and costs?	10. Can the results be applied to your local population/in your context?	11. Would the experimental intervention provide greater value to the people in your care than any of the existing interventions?
Berry	Yes	Yes	Yes	No	No	No	Yes	Yes	Yes	Yes	No	Cannot tell	Cannot tell

**Search Strategy Used:** (“vaccine administration” OR “vaccination administration” OR “vaccination program*” OR “vaccine program*” OR implementation OR rollout OR “roll out” OR distribution OR “delivery system*” OR “service delivery”) AND (“COVID-19 Vaccines”[Mesh] OR “COVID-19 vaccin*”[tiab] OR “COVID-19 immuniz*”[tiab] OR “COVID-19 immunis*”[tiab] OR “COVID-19 virus vaccin*”[tiab] OR “COVID-19 virus immuniz*”[tiab] OR “COVID-19 virus immunis*”[tiab] OR “COVID19 vaccin*”[tiab] OR “COVID19 immuniz*”[tiab] OR “COVID19 immunis*”[tiab] OR “coronavirus vaccin*”[tiab] OR “coronavirus immuniz*”[tiab] OR “coronavirus immunis*”[tiab] OR “SARS CoV 2 vaccin*”[tiab] OR “SARS CoV 2 immuniz*”[tiab] OR “SARS CoV 2 immunis*”[tiab]).
